# Brain tumours in the time of COVID-19: An online survey on patients’ disease experience in one Italian region

**DOI:** 10.3389/fonc.2023.1002895

**Published:** 2023-01-26

**Authors:** Giorgia Abete-Fornara, Francesca Mameli, Fabiana Ruggiero, Jennifer Meessen, Adriana Blanda, Antonella Ampollini, Marco Locatelli, Andrea Salmaggi, Andrea Di Cristofori, Ilaria Mauri, Manuela Caroli

**Affiliations:** ^1^ Department of Neuroscience and Mental Health, Foundation IRCCS Ca’ Granda Ospedale Maggiore Policlinico, Milan, Italy; ^2^ Department of Cardiovascular Medicine, Institute for Pharmacological Research Mario Negri IRCCS, Milan, Italy; ^3^ Department of Neurology, “A. Manzoni Hospital”, Lecco, Italy; ^4^ Department of Mental Health, Neurosurgery Unit, Fondazione IRCCS San Gerardo dei Tintori, Monza, Italia; ^5^ Neurology Unit, Ospedale San Gerardo, Monza, Italy; ^6^ Department of Psychology, University of Milano-Bicocca, Milan, Italy

**Keywords:** brain tumour, survey, psychological impact, pandemic, COVID-19, neuro-oncology

## Abstract

**Background:**

Since the outbreak, in 2019, of COVID-19, the world has experienced marked changes in daily habits, partly reflecting the exceptional social restrictions and health measures adopted to contain the disease. All these measures significantly affected not only peoples’s daily lives and psychological well-being but also the possibility for the healthcare system to function properly. In this setting, brain tumour patients were at risk due to their higher physical and mental fragility and their need for regular care. The aim of the present study was to assess, using a self-reported online questionnaire, the patients’s perceptions regarding their disease experience.

**Materials and methods:**

We developed an online anonymous self-report survey to assess patients’s disease experience during the pandemic. We investigated the impact of the COVID-19 pandemic on patients’s cancer care schedules, their psychological distress and emotions felt during the pandemic, their levels of worry about COVID-19, and their oncological conditions.

**Results:**

107 patients answered our survey, most of them suffering from a glioma. Less than one-third of the sample had their appointments cancelled, delayed or converted into online visits due to the pandemic. Of the patients who answered the survey, 95% declared they were satisfied with their Institute’s oncological management. The feelings reported most often were peacefulness or anxiety/worry; the majority of the sample reported high levels of loneliness, which tended to increase with age, whilst the psychological distress was correlated with age and with having a recurrence of the disease. Half of the sample declared severe worry about their oncological condition, in particular subjects with a recurrence or who were receiving adjuvant therapies. Patients with recurrence tended to worry more about the possibility of contracting COVID-19, and its effects.

**Conclusion:**

Our findings illustrate how fragile and in need of care patients with a brain tumour may be, especially those with more severe clinical conditions. These data may help boost healthcare professionals’s knowledge about brain tumour patients’s needs and fears, so as to be able to offer them a better hospital experience and improve their clinical management, while possibly also reducing the psychological burden on patients and their families.

## 1 Introduction

The 2019 coronavirus disease (COVID-19) first emerged in Wuhan, China, in late 2019 and then spread worldwide at the beginning of 2020. Italy was one of the most affected countries from February 2020 onwards and is still managing the pandemic waves with exceptional measures, such as lockdowns, quarantines, social restrictions, COVID-19 certifications, and vaccines. All of these measures have had significant effects not only on peoples’s daily lives but have also had an impact on the healthcare system’s ability to function properly and efficiently (1). For example, as of the end of January 2022, more than 19,600 patients with COVID-19 infection were hospitalized in Italy, greatly reducing the number of non-COVID-19 hospital beds available.

In fact, last winter, we experienced another shortage of hospital beds and delays in medical and surgical procedures, due to the increased need for COVID-19 spaces to cope with the fourth pandemic wave (December 2021-February 2022).

As previously reported, the repeated lockdowns, social restrictions, the sense of loneliness, and financial uncertainties, together with the fear of contracting the COVID-19 disease, have already caused physical and psychological distress in the general population (2, 3), leading to a higher risk of developing depressive, post-traumatic, and anxiety symptoms.

In this setting, patients with life-threatening conditions, such as brain tumours (low and high-grade gliomas -LGG and HGG-, meningiomas, and brain metastasis), are at risk on account of their greater physical and mental fragility, and their need for urgent care, which may be delayed because of the rising number of COVID-19 hospitalizations. Brain tumour patients frequently require medical visits and exams to monitor their oncological condition and to receive eventual therapies; this is especially the case for HGG patients who need adjuvant treatments after surgery. Moreover, they are known to suffer frequent mental health symptoms or disorders (for a recent systematic review see (4)), especially if a significant shift in people’s behaviours and social relations in response to the pandemic restrictions has led to changes in the possibility of patients’s families and caregivers to look after them.

All of these elements may have influenced patients’s disease experience, affectedpractical schedules (exams, visits, hospitalizations), and emotional and psychological reactions, including a higher risk to develop mood and anxiety disorders. It has already been reported how important social and family support is for brain tumour patients in order to prevent post-traumatic stress and reduce psychosocial distress (5, 6).

The aim of the present study was to assess, through a self-reported online questionnaire, the psychological and practical impact of the COVID-19 pandemic on the oncological care of patients with brain tumours.

Our findings may help boost healthcare professionals’s knowledge about brain tumour patients’s needs and fears, in order to offer thembetter hospital experiences and to improve their clinical management, while possibly also lowering the psychological burden on patients and their families.

## 2 Materials and methods

### 2.1 Procedure and questionnaire

We developed an anonymous self-report online survey in order to assess several points concerning brain tumour patients’s disease experience during the pandemic period (from March 2020, when COVID-19 spread through Italy, until January 2022, when our data collection ended). The questionnaire was assembled by our research team of psychologists and neuro-oncologists, taking inspiration from the current literature concerning brain tumours and COVID-19 data. Responses were collected over a period of 6 months (August 2021-January 2022). The questionnaire was administered through an online survey platform, which participants accessed using a dedicated link e-mailed by our collaborators. To reach the largest possible number of participants, it was also published on our institution’s web site and on our specialized centre of Neuro-oncology social media,. So as not to limit the data collection only to our patients, we presented the project to other neurosurgical or neuro-oncological units in other institutes in our region (Lombardy), where some of the most important centres for neuro-oncology are located and where the pandemic first spread in 2020 with the most significant impact. With this, we aimed to reach a wider and different sample of patients who might have experienced different oncological management from patients in our centre. The appropriate Ethics Committee reviewed and approved the survey distribution for this study, in accordance with local legal standards and authorized disclosure.

The survey could be answered by the patients themselves or by their caregivers, referring to the patient’s experience; it comprised 57 questions, with an estimated answering time of 5-10 minutes.

Answers were proposed on a Likert scale, from 1=not agree, to 4=completely agree.

The questionnaire was divided into the following areas:

- Sociodemographic data: age, sex, educational level, occupational status, living area, area of the institution where they are under treatment, and whether they live alone or not.
- Clinical data: kind of diagnosis, eventual ongoing adjuvant treatments, year of first diagnosis, and current clinical situation (first diagnosis or recurrence).
- COVID-19- related data: whether they contracted the virus or not, and whether they had received the vaccine.
- The impact of COVID-19 on cancer care, as described by Kosi (7): *respondents self-reported if and how the pandemic-related events disrupted their cancer treatment and care, if they received information about the novel SARS-CoV-2, and how satisfied they were with the information available*. We directly investigated changes and disruptions in oncological care due to the pandemic, such as conversions into online modalities.
- The sense of loneliness due to social restrictions to prevent the further spread of the virus. To assess this construct, we used the De Jong Gierveld questionnaire, a six-item questionnaire to detect emotional and social loneliness (8), together with other questions of interest, based on Kosir’s definition: *mentions of lacking support, feeling low and sad because of isolation, feelings of loneliness (* 7 *)*.
- The general psychological distress related to the pandemic period was measured with the Patient Health Questionnaire for Depression and Anxiety (PHQ-4 (9)), a four-item questionnaire, to briefly detect levels of anxiety and depression.
- Worry about COVID-19, a construct previously described in the literature as “*worries about how one’s body would react to COVID-19, worries about severe complications due to COVID-19, including worries about receiving adequate care if one were to contract COVID-19” (* 7 *)*.
- Worry about neuro-oncological conditions: fears related to the medical conditions were addressed in several questions inspired by an open-access online survey for oncological patients promoted by AIMAC (Italian Association of cancer patients).
- Emotional status and coping strategies implemented to cope with them: emotions and feelings experienced during the pandemic months, regarding the oncological diagnosis, was measured using a frequency Likert scale, from 1=never or almost never, to 4=always or very often. We proposed a list of several emotions, feelings, and coping strategies, inspired by an open-access online survey for oncological patients promoted by AIMAC (Italian Association of cancer patients).

During the survey, several indications and instructions guided the participants to answer referring to their specific experience during the pandemic months, thus avoiding consideration of eventual previous different experiences. For example, information concerning psychological health and well-being experienced by patients before their oncological diagnosis can be difficult to assess and investigate through an online patient-reported questionnaire because of the possible time delay between diagnosis and the answering of the questionnaire. Another factor are the possible difficulties faced by patients or caregivers to identify psychological distress. For example, those with a low educational level or those who refuse to admit that they might suffer from psychological distress even in the presence of symptoms. Moreover, from a clinical point of view, it can be very difficult to distinguish psychological symptoms generated by the tumour itself, especially in the case of frontal or medial-temporal lesions, from those caused by stressful life events of the patient, meaning from non-organic origins). To avoid interference from previous psychological symptoms on the survey answers, we strongly indicated in several points of the questionnaire that the answers to the questions should refer only to those feelings and symptoms that emerged and were felt during the pandemic months and, depending on the survey section and related construct, were related to the patient’s oncological situation or to the COVID-19 pandemic. In this way, we tried to guide patients to answer our survey thinking about the specific psychological attitude that emerged during the pandemic, thus excluding eventual pre-existing symptoms.

### 2.2 Participants

Inclusion criteria were: (i) over 18 years old and, (ii) diagnosed with a brain tumour (LGG, HGG, meningiomas, metastasis) before January 2022, when the online survey was closed. All participants voluntarily answered the anonymous questionnaire and gave their informed consent to participate. The aims were broadly described, and patients could interrupt and quit the survey at any point.

### 2.3 Statistical analysis

Descriptive statistics were performed in order to describe our sample and the constructs presented above.

Multinomial logistic regression analyses were run to investigate the associations between a series of continuous and categorical predictors (socio-demographical and clinical data) and our constructs and responses to the survey questions.

Statistical analyses were performed with SPSS Statistics 26 (IBM SPSS Statistics, New York, NY). The level of statistical significance was set at a *p-*value < 0.05.

## 3 Results

### 3.1 Sociodemographic and clinical data

A total of 108 patients participated in our survey, including a 5-year-old male child with an LGG and whose questionnaire was completed by a parent; however, he was excluded from statistical analyses as he was considered an outlier and was only described qualitatively. Thus, the final sample analysed consisted of 107 patients, composed of 58 (54,2%) women and 49 (45,8%) men, with a mean age of 54,1 years (SD 12,7), ranging from 23 to 81. Concerning the level of education, almost half of the sample (51 patients, 47.7%) had a high school diploma and a full-time job (N=50, 46,7%). As for their living situation, 97 patients lived with relatives whilst only 10 patients (9,3%) declared to live alone.

As regards diagnosis, patients with a glioma represented the majority of the sample (81 patients): 45 patients (42,1%) had an HGG and 36 (33,6%) an LGG; 76 patients (71%) had only a first diagnosis, whilst 31 (29%) experienced a recurrence. A total of 67 patients (62,6%) said they were not receiving adjuvant treatments (chemotherapy and/or radiotherapy) at the time of answering the questionnaire.

The survey was completed by the patients themselves in 84 cases (78,5%) and by a caregiver in 22 cases (20,56%). Complete sociodemographic and clinical data of the 107 patients are reported in **Table 1**.

**Table 1:** complete list of sociodemographic and clinical data of the sample.

Age: mean (SD); range	54,06 (12,73); 23-81
Educational Level:	Elementary School= 6 (5,6%) Middle School= 18 (16,8%) High School= 51 (47,7%) Degree = 25 (23,4%) PhD or MSC = 7 (6,5%)
Gender: N (%)	Male = 49 (45,8%) Female= 58 (54,2%)
Working status: N (%)	Homeworkers= 6 (5,6%) In search for a job= 3 (2,8%) Part-time worker= 10 (9,3%) Full-time worker= 50 (46,7%) Retired= 37 (34,6%) Students= 1 (0,9%)
Live alone: N (%)	Yes= 10 (9,3%) No= 97 (90,7%)
Diagnosis: N (%)	LGG= 36 (33,6%) HGG= 45 (42,1%) Meningioma= 21 (19,6%) Metastasis= 5 (4,7%)
Actual clinical situation: N (%)	First Diagnosis= 76 (71%) Recurrence= 31 (29%)
Adjuvant therapies in progress: N (%)	Yes= 40 (37,4%) No= 67 (62,6%)
Contracted the COVID-19 virus: N (%)	Yes= 6 (5,6%) No= 101 (94,4%)
Already received at least one dose of the COVID-19 vaccine: N (%)	Yes= 105 (98,1%) Not yet= 2 (1,8%)

SD, Standard Deviation; N, number; LGG, low-grade glioma; HGG, high-grade glioma.

### 3.2 Impact of COVID-19 on cancer care

Only six patients (5,6%) contracted the COVID-19 virus at the time of answering the questionnaire. Patients declared to have been supported adequately for their oncological condition: 95,3% responded that they had received adequate indications concerning the oncological management during the pandemic. However, only 59,8% of the patients received enough information on how to manage the pandemic while being a fragile subject.

Due to the significant impact of the pandemic on hospital management, we investigated whether medical teams delayed scheduled visits or examinations: 29,9% of the population had appointments cancelled, and 28,9% experienced delays. For 21,5% of the patients, the medical team proposed converting visits into online forms, such as video call, emails, and messages.

To avoid going to hospitals, 21,5% of the patients themselves cancelled scheduled appointments and 17,8% asked to convert their appointments into online visits. Besides these concerns, almost all patients (95,3%) stated they were sufficiently or completely satisfied with their medical teams’s general clinical management during the pandemic.

### 3.3 Psychological functioning

We asked our patients to rate the frequency of certain emotions and feelings experienced during the pandemic, referring to their neuro-oncological condition. Interestingly, peacefulness and anxiety/worry were the two most frequently reported: the former with 57 patients (53,3%) declaring to feel it more than half of the time or always, the latter with 45 (42,1%) declaring the same. The two emotions that were felt the least were indifference, with only 14 patients (13,1%) experiencing it frequently, and anger, with 22 patients (20,5%).

Other feelings investigated were fear, confusion, and sadness, with respectively 43 (40,2%), 34 (31,8%), and 38 (35,5%) patients declaring to have felt these at least more than half of the time. Concerning the sense of loneliness, measured through the questionnaire cited above, only five patients claimed not to have felt lonely (4,7%), whilst 70 (65,4%) felt moderately lonely, and 32 (29,9%) felt severely lonely. Furthermore, the general psychological distress resulted in clinically severe distress only in 13 patients (12,1%); moderate distress emerged for 16 (15%), mild for 28 (26,2%), and 50 patients (46,7%) reported no psychological distress.

### 3.4 Coping strategies

We investigated the coping strategies most frequently reported in the literature, in order to understand how our patients managed their emotions and feelings during this period. We proposed a list of practical strategies easily accessible at home, in consideration of the COVID-19 restrictions.

The most popular strategy turned out to be phoning friends and relatives, with 52 patients (48,6%) using it at least half of the time or always; the second strategy involved using social media or apps, with 25 patients (23,4%) using web resources frequently. Hardly anyone said they had contacted other patients with the same diagnosis or caregiver associations, with only 2 (1,9%) and 3 patients (2,8%) having resorted to these options, respectively. Asking for professional psychological help or practising meditation or mindfulness techniques was also infrequent: 16 (15%) and 12 (11,2%) patients, respectively. No other strategies were taken into account by our patients: 80 patients out of 107 declared they did not use any other tool.

### 3.5 Worry about brain tumour

During the pandemic, moderate or severe worry (at least a score of 12 out of 16 on the global construct) emerged in 28,8% of the sample. Specifically, 50,5% of the sample felt very worried about their clinical condition and 54,2% felt more fragile and vulnerable than the healthy population. The possibility of delays and disruptions in oncological procedures was a matter of concern for 56% of the sample, whilst 72,9% felt sufficiently comfortable about going to hospitals for visits and examinations.

### 3.6 Worry about COVID-19

We used the “worry about COVID-19” construct to verify the level of worry about possibly being at a greater risk of contracting the disease or developing a more severe form in case of infection because of the patient’s oncological condition,. The answers ranged between great worry (43.9%) and a low level of concern (31.8%), with 24,3% declaring a neutral position towards these fears.

### 3.7 Assessing variables associated with psychological outcomes

In order to assess which variables were significantly associated with responses to the survey questions, multinominal logistic regression analyses were performed, including age, sex, diagnosis, adjuvant therapy, clinical status, and living situation.

#### 3.7.1 Psychological distress scale

For this outcome scale (represented by the PHQ-4), there was a significant effect of age: the probability of experiencing severe psychological distress was higher in older patients, compared to the absence of distress (OR 1.17, 95%CI 1.07-1.27, p=0.0004). Similar results, when taking age into consideration, were observed in the other levels of psychological distress (mild OR1.05, 95%CI 1.01-1.10, p=0.028; moderate OR:1.03 95%CI 0.97-1.08, p=0.350). In addition, compared to normal levels, those patients with severe psychological distress had seven times higher odds of having had a recurrence instead of a first diagnosis. None of the other variables included were associated with specific levels of psychological distress; for the complete list of results, see **Table 2**.

**Table 2:** Variables associated with psychological distress-Results of multinomial regression analysis.

Ref= normal		OR	95% confidence interval		P
**mild**	**Age**	**1.053**	**1.006**	**1.102**	**0.028**
mild	Sex (female)	0.646	0.230	1.816	0.407
mild	Diagnosis: Lgg vs meningioma	1.307	0.313	5.457	0.713
mild	Diagnosis: Hgg vs meningioma	1.779	0.460	6.882	0.404
mild	Diagnosis: metastasis vs meningioma	1.616	0.072	36.440	0.762
mild	Adjuvant therapies (yes)	0.984	0.321	3.010	0.976
mild	Clinical Status (recurrence)	2.237	0.719	6.965	0.164
mild	Not living alone	1.257	0.234	6.756	0.790
moderate	Age	1.025	0.973	1.081	0.350
moderate	Sex (female)	0.935	0.261	3.353	0.917
moderate	Diagnosis: Lgg vs meningioma	4.131	0.375	45.529	0.246
moderate	Diagnosis: Hgg vs meningioma	5.297	0.538	52.184	0.153
moderate	Diagnosis: metastasis vs meningioma	18.762	0.746	471.981	0.074
moderate	Adjuvant therapies (yes)	0.476	0.126	1.804	0.275
moderate	Clinical Status (recurrence)	1.445	0.355	5.888	0.607
moderate	Not living alone	0.988	0.082	11.865	0.992
**severe**	**Age**	**1.168**	**1.072**	**1.272**	**0.0004**
severe	Sex (female)	1.622	0.350	7.513	0.536
severe	Diagnosis: Lgg vs meningioma	6.877	0.522	90.532	0.142
severe	Diagnosis: Hgg vs meningioma	6.548	0.503	85.229	0.151
severe	Diagnosis: metastasis vs meningioma	3.535	0.067	186.115	0.532
severe	Adjuvant therapies (yes)	0.442	0.077	2.531	0.358
**severe**	**Clinical Status (recurrence)**	**7.887**	**1.495**	**41.604**	**0.014**
severe	Not living alone	0.772	0.057	10.458	0.845

Bold data represent significant results (p < 0.05).

#### 3.7.2 Sense of loneliness

Age had a significant effect on loneliness: compared with those who had a severe sense of loneliness, those who experienced less loneliness were younger (moderate: OR=0.95, 95%CI 0.91-0.99, p=0.008; not lonely 0.96 95%CI 0.88-1.05, p=0.333). None of the other variables included were significantly associated with specific levels of sense of loneliness, although there was a non-significant trend for women to have higher odds of feeling lonely more severely. For the complete list of results, see **Table 3**.

**Table 3:** – Variables associated with Sense of Loneliness- Results of multinomial regression analysis.

Ref=severely		OR	95% confidence interval		P
**moderately**	**Age**	**0.948**	**0.911**	**0.987**	**0.008**
moderately	Sex (female)	1004	0.383	2.630	0.993
moderately	Diagnosis: Lgg vs meningioma	0.775	0.198	3.041	0.715
moderately	Diagnosis: Hgg vs meningioma	0.975	0.263	3.619	0.969
moderately	Diagnosis: metastasis vs meningioma	0.445	0.049	4.052	0.472
moderately	Adjuvant therapies (yes)	1.139	0.400	3.247	0.807
moderately	Clinical Status (recurrence)	2.105	0.703	6.307	0.183
moderately	Not living alone	1249	0.263	5.946	0.779

Bold data represent significant results (p < 0.05).

#### 3.7.3 Worry about brain tumour

For this variable, the clinical status and the presence of adjuvant therapies had significant effects: the most important predictor for greater worry about the tumour (as compared to low worry) was whether the patient was receiving adjuvant therapies (neutral tumour worry OR=5.26, 1.60-17.30, p=0.006; severe tumour worry OR=5.41, 95%CI 1.59-18.41, p=0.006). In addition, compared to those with low tumour worry, those with severe tumour worry had four times the odds of having had a recurrence instead of a first diagnosis (OR=4.25, 95%CI: 1.33-13.56, p=0.014); for the complete list of results, see **Table 4**.

**Table 4:** Variables associated with Worry about tumour- Results of multinomial regression analysis.

Ref = low		OR	95% confidence interval		P
neutral	Age	1.011	0.969	1.056	0.605
**neutral**	**Sex (female)**	**3.259**	**1.046**	**10.152**	**0.041**
neutral	Diagnosis: Lgg vs meningioma	0.345	0.071	1.684	0.188
neutral	Diagnosis: Hgg vs meningioma	0.736	0.222	2.441	0.318
neutral	Diagnosis: metastasis vs meningioma	0.346	0.023	5.123	0.998
**neutral**	**Adjuvant therapy (ref=no)**	**5.255**	**1.597**	**17.298**	**0.006**
neutral	Clinical Status (recurrence)	0.462	0.143	1.488	0.195
neutral	Not living alone(ref=no)	2.077	0.292	14.764	0.465
**severe**	**Age**	**1.062**	**1014**	**1.113**	**0.011**
severe	Sex (female)	1.955	0.649	5.886	0.233
severe	Diagnosi Igg vs meningioma	0.308	0.063	1.507	0.146
severe	Diagnosi Hgg vs meningioma	0.774	0.231	2.590	0.230
severe	Diagnosi metastasis vs meningioma	0.432	0.039	4.765	0.786
**severe**	**Adjuvant therapies (no)**	**5.411**	**1.591**	**18.409**	**0.006**
**severe**	**Clinical Status (recurrence)**	**0.235**	**0.074**	**0.752**	**0.014**
severe	Not living alone	2.303	0.388	13.673	0.358

Bold data represent significant results (p < 0.05).

#### 3.7.4 Worry about COVID-19

For COVID-related concerns, sex and clinical status showed significant effects: women had more probability of showing a low level of worry than men (OR=4.39, 95%CI 1.49-12.92, p=0.007). In addition, in the severe worry category, there were very low odds that the patient had a first diagnosis rather than a recurrence (OR=0.31, 95%CI 0.10-0.98, p=0.046). For the complete list of results, see **Table 5**.

**Table 5:** – Variables associated with Worry about COVID-19- Results of multinomial regression analysis.

ref=low		OR	95% confidence interval		P
neutral	Age	0.997	0.955	1.041	0.906
neutral	Female	2.601	0.817	8.282	0.105
neutral	Igg vs meningioma	0.675	0.141	3.242	0.623
neutral	Hgg vs meningioma	1.541	0.436	5.447	0.502
neutral	metastasis vs meningioma	1.671	0.077	36.192	0.743
neutral	Adjuvant therapy (ref=no)	0.816	0.231	2.883	0.752
neutral	*Clinical Status (recurrence)*	0.818	0.220	3.041	0.764
neutral	Not living alone(ref=no)	3.104	0.396	24.366	0.281
severely	Age	1.022	0.981	1.066	0.296
**severely**	**Female**	**4.392**	**1.493**	**12.916**	**0.007**
severely	Igg vs meningioma	0.739	0.175	3.119	0.680
severely	Hgg vs meningioma	2.060	0.638	6.651	0.226
severely	metastasis vs meningioma	2.351	0.177	31.264	0.517
severely	Adjuvant therapy (ref=no)	2.260	0.736	6.945	0.154
**severely**	***Clinical Status (recurrence)***	**0.310**	**0.098**	**0.982**	**0.046**
severely	Not living alone(ref=no)	4.931	0.698	34.849	0.109

Bold data represent significant results (p<0.05).

## 4 Discussion

This survey inquired how brain tumour patients dealt with the pandemic period in terms of practical and psychological experiences related to their oncological pathways.

Concerning the practical influences of pandemic-related measures on clinical management, our results indicated that patients followed by Lombardy institutes were generally well satisfied with the support and clinical management received by their medical team during the pandemic months. This is a positive finding that underlines the Lombardy healthcare system’s successful effort to preserve fragile patients even during difficult periods such as the pandemic. Nevertheless, only approximately half of the sample declared they had received enough information about how to manage, as a more fragile category, their daily routine and clinical follow-ups. Unsatisfactory communication and lack of information tailored to their being oncological patients were already reported (7).Thus, the need for specific and tailored information should be considered by clinicians in the future.

In the period and region considered, scheduled appointments for visits and exams were rarely cancelled or delayed, even by hospitals or patients themselves; this latter result may be explained by the patients’s low levels of fear about attending hospitals even though they were places with increased risks to contract COVID-19, especially in the first wave of the pandemic (March-May 2020). Interestingly, this is in contrast with what emerged from an international survey by Voisin and colleagues (10),who found that European patients, during 2020, suffered the most from appointment delays, compared to other continents. Moreover, patients rarely had or asked to have their appointments converted to online modalities, such as video calls: this might be explained by the fact that brain tumour patients need radiological follow-ups or therapeutic sessions which cannot be converted to online appointments. Our Institute set up dedicated protected pathways during lockdowns for oncological visits, so as not to delay the fixed schedules patients had to follow (11); also the use of emails to contact the medical team was already established before the virus outbreak.

Concerning the psychological impact and reactions of patients related to their oncological status during the pandemic, the present study found how patients differed in their emotions and feelings: peacefulness and anxiety-worry were the two emotions felt most often, describing a population that approaches the oncological condition from two opposite psychological perspectives. Indifference and anger towards future and oncological developments were rarely reported, whilst sadness and fear were reported by less than half of the sample. In a recent international study about the impact of the pandemic on oncological patients (12), fear emerged as the predominant emotion reported, especially related to possible delays in treatments or a possible COVID-19 infection. Although these concerns were expressed in our sample, they led to anxiety more than fear.

Approximately half of the sample did not experience clinically significant psychological distress, defined as anxiety or depressive symptoms, although our results showed that patients with a tumour recurrence tended to report higher levels of psychological distress, as did elderly patients. This was a coherent finding, as these two classes of patients are faced with greater concerns and difficulties in their daily life and have to rely on caregivers more than younger or healthier people, especially during the pandemic. The relative absence of severe anxiety and depressive symptoms in brain tumour patients already emerged in literature during the pandemic (6), although in contrast with other studies that found high rates of depressive and anxiety symptoms among cancer patients (13).

Concerning the sense of loneliness, we found a slight trend for women to feel more lonely than men and a tendency for this to increase for elderly people, a result that echoes reports in the general population concerning psychological well-being during the COVID-19 outbreak (14, 15). The association between age and the psychological impact of the pandemic has led to controversial results in the literature: both young and elderly people have been described as those suffering the most (14, 16, 17). As reported by Cheng and colleagues (18), the younger population has greater access to web resources, which may have been a positive resource during lockdowns, helping in managing psychological distress and loneliness, instead of triggering anxiety as suggested by the authors; at the same time, elderly patients may have suffered more from social restrictions and isolation. High levels of sense of loneliness, as those found in our study, were already described in the general oncological population during the pandemic (13) and were also associated with depressive symptoms (19), highlighting how widespread and significant this feeling is among cancer patients.

To cope with psychological distress or with negative emotions, our survey found a limited range of resources implemented. Besides, Lombardy is a region with easy access to several services and possibilities: our sample of patients declared to rely mainly on friends and family support, and also through phone calls when social restrictions forbade personal contacts. This evidence was in line with previous results in the body of literature (6, 10),which found how important social support is for brain tumour patients in terms of quality of life. Social media and web resources were a secondary strategy employed to achieve support and distraction, whilst psychological help, associations of patients or relatives, and meditative techniques were seldom or never addressed by our sample. To our knowledge, in Italy there are no active associations for brain tumour patients or relatives.

Oncological patients had worries about contracting the virus, and the survey indicated an association between levels of worry and gender or diagnosis, with women or recurrence patients feeling more worried. In a previous international survey (10), no association was found between clinical status or other sociodemographic data and levels of worry about COVID-19, although almost half of that sample declared to perceive higher risks in case of infection. This result, similar to ours, stresses how the COVID-19 disease has affected psychological well-being.

Furthermore, in our research, half of the sample felt severely worried about their oncological condition and more fragile than the healthy population, a perception already described in general oncological patients (20). Similarly, more than half of our respondents expressed concerns about possible delays in scheduled clinical appointments and this concern was one of the most intense and frequently reported, data in line with previous findings concerning brain tumour patients worldwide (10, 12). A diagnosis of recurrence and attending chemotherapy or radiotherapy when answering the survey were associated with higher levels of concern about clinical conditions, illustrating the impact of the COVID-19 pandemic on fragile peoples’s daily lives and well-being.

Of course, patients with a more severe clinical condition or the elderly population may suffer from clinical deterioration related to various factors determined by their disease, such as the site of the tumour or the side effects of adjuvant therapies. Nevertheless, this survey was conceived anonymously, therefore it was difficult to distinguish how the objective aspects concerning patient general status prevail over the emotional components (concern for one’s health). Certainly, both components may have had moderate relevance as confirmed by the results, which showed how patients with a more severe clinical status expressed greater concerns about their oncological condition and lower levels of psychological well-being in general. It can be considered that adjuvant therapies, older age, or recurrence are clinical elements that, due to their personal, clinical, and social consequences, led to poorer psychological well-being and greater concerns. However, with this methodology, we wanted to focus on the patients’s points of view concerning their perceptions of health conditions during the COVID pandemic, as affected by a neuro-oncological pathology. This was why, although appropriate, it might have been difficult to determine the exact relevance of the single parameters cited above, in particular in the elderly population.

## 5 Conclusion

This survey shows how brain tumour patients are at great risk of severe psychological impact and distress, especially those with a recurrence or who are receiving adjuvant therapies or who are elderly. In these groups, the COVID-19 pandemic increased worries and concerns about current and future conditions, although without causing widespread significant psychopathology.

Our results depict indications for clinicians to consider in the future: more attention should be dedicated to providing better and more specific information for patients and their families about their medical conditions and how to manage them during a pandemic. Furthermore, this study also highlights the importance of preserving protected pathways for fragile patients so as not to delay diagnostic or therapeutic appointments, which was one of the patients’s main concerns, even during difficult periods, such as the current pandemic.

## 6 Limitations

The most significant limitation of our study was represented by the distribution of the sample, which was impossible to control given the voluntary participation recruitment method. For example, the overwhelming majority of the patients had not contracted COVID-19 and almost all were vaccinated; moreover, a diagnosis other than glioma was not comparable because of the significantly smaller numbers. These elements may have affected patients’s behaviour, but were not possible to study. Therefore, statistical analyses - and consequently also discussion of the results - were limited to the other categories. This prevented a more detailed analysis of the data and meant we could not study all categories of patients and constructs.

Secondarily, the sample described belonged to a single Italian region, thus reported practical experiences referred to several Institutes located in a restricted area of Italy and may not represent those of other regions and areas. In this view, our results should not be generalised to the whole nation.

## Data availability statement

The original contributions presented in the study are included in the article/supplementary material. Further inquiries can be directed to the corresponding author.

## Ethics statement

The studies involving human participants were reviewed and approved by Foundation IRCCS Ca’ Granda Ospedale Maggiore Policlinico, 20122 Milan, Italy. The patients/participants provided their written informed consent to participate in this study.

## Author contributions

A-FG studied design and methodology, data analysis, designed tables, and wrote the draft; MJ and BA performed the statistical analysis and designed tables; MF and RF studied design and methodology, and reviewed the final manuscript; SA, DA and MI contributed in spreading the survey; CM studied design and reviewed the final manuscript; AA and LM reviewed the final manuscript. All authors contributed to the article and approved the submitted version.
