# Erratum: Brain tumours in the time of COVID-19: An online survey on patients’ disease experience in one Italian region

**DOI:** 10.3389/fonc.2023.1187714

**Published:** 2023-03-27

**Authors:** Frontiers Production Office

**Affiliations:** Frontiers Media SA, Lausanne, Switzerland

**Keywords:** brain tumour, survey, psychological impact, pandemic, COVID-19, neuro-oncology

Due to a production error, the given names and surnames of the authors in the author list were reversed. The author list was mistakenly published as “Abete-Fornara Giorgia^1^, Mameli Francesca^1^, Ruggiero Fabiana^1^, Meessen Jennifer^2^, Blanda Adriana^2^, Ampollini Antonella^1^, Locatelli Marco^1^, Salmaggi Andrea^3^, Di Cristofori Andrea^4^, Mauri Ilaria^5^,^6^ and Caroli Manuela^1^*”. The correct author list is “Giorgia Abete-Fornara^1^, Francesca Mameli^1^, Fabiana Ruggiero^1^, Jennifer Meessen^2^, Adriana Blanda^2^, Antonella Ampollini^1^, Marco Locatelli^1^, Andrea Salmaggi^3^, Andrea Di Cristofori^4^, Ilaria Mauri^5^,^6^, Manuela Caroli^1^*”. The publisher apologizes for this mistake.

The original version of this article has been updated.

